# Clinicopathological Characteristics and BAP1 Expression in an Enucleation-Based Uveal Melanoma Cohort: A Single-Center Croatian Experience with Long-Term Follow-Up

**DOI:** 10.3390/cancers18081211

**Published:** 2026-04-10

**Authors:** Domagoj Vlašić, Mira Knežić Zagorec, Antonia Jakovčević, Dina Lešin Gaćina, Marijana Ćorić, Tomislav Jukić

**Affiliations:** 1 Department of Ophthalmology, Dubrovnik General Hospital, 20000 Dubrovnik, Croatia; 2 Department of Ophthalmology, University Hospital “Sveti Duh”, 10000 Zagreb, Croatia; 3 Department of Pathology and Cytology “Ljudevit Jurak”, University Hospital Centre “Sestre milosrdnice”, 10000 Zagreb, Croatia; 4 Department of Ophthalmology, University Hospital Centre Zagreb, 10000 Zagreb, Croatia; 5 Department of Pathology and Cytology, University Hospital Centre Zagreb, 10000 Zagreb, Croatia; marijana.coric@kbc-zagreb.hr; 6 School of Medicine, University of Zagreb, 10000 Zagreb, Croatia

**Keywords:** uveal melanoma, BAP1, immunohistochemistry, enucleation, survival, Croatia, selection bias, choroidal melanoma, tumor suppressor, descriptive study

## Abstract

Background/Objectives: Loss of nuclear BAP1 (BRCA1-associated protein 1) expression is a well-established adverse prognostic marker in uveal melanoma (UM). However, data from Central and Southeastern European populations are limited. This descriptive study aimed to evaluate BAP1 immunohistochemical expression in a Croatian enucleation-based UM cohort, characterize its associations with clinicopathological parameters, and contextualize the findings within the published literature. Methods: Formalin-fixed, paraffin-embedded tumor tissue from 58 consecutive patients with primary choroidal and ciliary body melanoma treated with enucleation at University Hospital Centre Zagreb (2006–2016) was analyzed immunohistochemically for BAP1 nuclear expression. Associations with clinicopathological parameters were assessed using chi-square and Fisher’s exact tests. Survival analysis was performed using Kaplan–Meier estimation, log-rank tests, and Cox proportional hazards regression with a median follow-up of 11.2 years. Results: Loss of nuclear BAP1 expression was observed in 53/58 (91.4%) specimens, resulting in a severely imbalanced distribution (53 versus 5 patients) precluding meaningful comparative survival analysis. Five-year and 10-year overall survival rates were 72.4% and 51.7%, respectively, with a median overall survival of 14.5 years. BAP1 loss was associated with longer disease-free survival (log-rank *p* = 0.020); however, this finding likely reflects a statistical artifact attributable to the extremely small BAP1-retained group (*n* = 5) harboring concurrent adverse features and should not be interpreted biologically. The study was underpowered to draw prognostic inferences regarding BAP1 status. Exploratory survival analyses are presented for transparency but should not be interpreted inferentially. Conclusions: The exceptionally high prevalence of BAP1 loss reflects the selection bias inherent in enucleation-based cohorts, which are enriched for large, molecularly high-risk tumors. This study provides the first comprehensive BAP1 immunohistochemical data from Croatia, contributing to the growing evidence that enucleation cohorts represent a distinct, biologically high-risk subgroup in which BAP1 immunohistochemistry offers limited discriminatory value. The extended follow-up of 11.2 years confirms the prolonged natural history of UM. Future multi-center studies incorporating molecular validation and diverse treatment modalities are needed to establish the prognostic utility of BAP1 across the full spectrum of UM disease.

## Simple Summary

Uveal melanoma is the most common primary eye cancer in adults and spreads to the liver in about half of patients, leading to poor outcomes. The protein BAP1, which normally suppresses tumor growth, is frequently lost in aggressive uveal melanomas. However, data on BAP1 loss in Central and Southeastern European populations are scarce. In this descriptive study, we analyzed BAP1 protein expression in tumor tissue from 58 Croatian patients who underwent surgical removal of the eye for uveal melanoma and were followed for a median follow-up of over 11 years—one of the longest follow-up periods reported. We found BAP1 loss in 91.4% of patients, substantially higher than typical literature values of 43–56%, which we attribute to the advanced nature of tumors requiring eye removal. The severely imbalanced distribution of BAP1 status (53 versus 5 patients) precluded meaningful prognostic analysis. These findings provide the first comprehensive BAP1 immunohistochemical data from Croatia and highlight the critical impact of study design on the reported prevalence of BAP1 loss.

## 1. Introduction

Uveal melanoma (UM) is the most common primary intraocular malignancy in adults, with an annual incidence of 5–7 cases per million in Western populations [1,2]. Despite high rates of local tumor control exceeding 95% with contemporary treatment modalities, the 10-year metastatic rate remains approximately 34–50%, with the liver as the predominant site of dissemination [3,4]. Once metastatic disease develops, the prognosis is poor, with a median survival of 6–12 months and limited response to currently available systemic therapies [5]. The recent approval of tebentafusp for HLA-A*02:01-positive patients represents the first significant advance in metastatic UM, yet the overall survival benefit remains modest (median 21.7 vs. 16.0 months) [6].

The identification of inactivating mutations in the *BAP1* gene (BRCA1-associated protein 1, chromosome 3p21.1) by Harbour et al. in 2010 represented a paradigm shift in our understanding of UM biology [7]. *BAP1* encodes a nuclear deubiquitinase that removes monoubiquitin from histone H2A at lysine 119 (H2AK119ub1) as the catalytic subunit of the Polycomb repressive deubiquitinase (PR-DUB) complex [8]. This activity maintains open chromatin configurations at promoters of melanocyte differentiation genes, including *MITF*, *TYR*, and *DCT*, thereby preserving a differentiated, non-metastatic phenotype [9]. Loss of BAP1 function, occurring through biallelic inactivation (typically monosomy 3 combined with a point mutation on the remaining allele), results in chromatin compaction, transcriptional silencing of differentiation programs, and acquisition of a primitive, stem cell-like phenotype associated with metastatic competence [10].

Immunohistochemical (IHC) detection of BAP1 loss has emerged as a practical and cost-effective prognostic tool for routine paraffin-embedded specimens [11,12,13,14]. Multiple studies have demonstrated that loss of nuclear BAP1 staining correlates strongly with monosomy 3, a gene expression class 2 (metastatic) phenotype, and adverse clinical outcomes [11,12,13,14,15,16,17]. The Liverpool group reported that BAP1 loss was present in 43% of their series of enucleation specimens and was independently associated with increased metastatic risk [11]. Koopmans et al. observed *BAP1* loss in 51% of a mixed cohort of 74 patients [12]. Most recently, Kennedy et al. analyzed 308 consecutive enucleations from Dublin and demonstrated that BAP1 loss outperformed AJCC staging, monosomy 3 status, and TCGA molecular classification in predicting survival [17].

Despite this extensive literature, data on BAP1 expression in UM from Central and Southeastern European populations remain scarce. Although a recent comprehensive analysis of 1140 primary UM tumors has characterized the molecular landscape across tumor sizes [18], most BAP1 IHC studies are based on enucleation cohorts, which are systematically enriched for large, advanced tumors with unfavorable molecular profiles. This selection bias is a critical consideration when evaluating the prevalence of BAP1 loss and its prognostic value. Epidemiological data on UM in Croatia are limited to a single retrospective analysis from Split-Dalmatia County, which reports an incidence of 4.4 per million [19,20].

This study aimed to: (1) determine the prevalence of BAP1 loss by IHC in a well-characterized Croatian UM cohort treated with enucleation; (2) assess associations between BAP1 status and clinicopathological parameters; (3) describe overall survival (OS) and disease-free survival (DFS) in this cohort and perform exploratory analyses of BAP1 status in relation to outcomes; and (4) contextualize our findings within the published literature, with particular attention to the influence of cohort selection on the reported prevalence of BAP1 loss.

## 2. Materials and Methods

### 2.1. Study Design and Patient Selection

This retrospective, single-center cohort study was conducted on archived formalin-fixed, paraffin-embedded (FFPE) tumor tissue specimens stored at the Department of Pathology and Cytology at the University Hospital Centre (UHC) Zagreb. A search of the institutional database of the Department of Ophthalmology identified 62 consecutive patients who underwent primary enucleation for histopathologically confirmed UM of the choroid and/or ciliary body between 1 January 2006 and 31 December 2016.

Inclusion criteria were: (i) histopathologically confirmed primary choroidal and/or ciliary body melanoma; (ii) adequate tumor tissue available in archived FFPE blocks; (iii) largest tumor diameter (LTD) measured before fixation; (iv) complete clinical data, including demographics and outcome; and (v) minimum follow-up of 5 years or documented death within the follow-up period.

Exclusion criteria were: (i) prior radiotherapy, chemotherapy, or any oncological treatment before enucleation; (ii) primary iris melanoma; (iii) inadequate tissue for IHC analysis (e.g., poor fixation, insufficient tumor volume); (iv) extensive tumor necrosis precluding evaluation; and (v) dense pigmentation interfering with IHC visualization.

After applying these criteria, four patients were excluded (two for inadequate fixation and two for extensive necrosis), yielding in a final study cohort of 58 patients.

Clinical and histopathological data were extracted from medical records at UHC Zagreb. Mortality data were obtained from the Croatian National Cancer Registry. OS was defined as the interval from enucleation to death from any cause or last follow-up. DFS was defined as the interval from enucleation to the first documented metastasis, death from UM, or last follow-up. The study was censored on 31 December 2023. The study protocol was approved by the Ethics Committee of UHC Zagreb (protocol no. 8.1-21/248-2, 02/21 AG; approved 29 November 2021) and conducted in accordance with the Declaration of Helsinki. Given the retrospective nature of the study, which used archived tissue, the Ethics Committee waived the requirement for individual informed consent.

### 2.2. Histopathological Evaluation

All hematoxylin and eosin-stained slides were reviewed by an experienced ophthalmic pathologist. Tumors were classified according to the 2025 World Health Organization (WHO) Classification of Tumours of the Eye as follows: (a) spindle cell melanoma—composed exclusively of spindle cells; (b) mixed cell melanoma—containing both spindle and epithelioid cells; and (c) epithelioid melanoma—comprising >50% epithelioid cells. The following parameters were recorded: LTD (mm), ciliary body involvement (present/absent), mitotic count (per mm^2^), lymphocytic infiltration (present/absent), scleral involvement (present/absent), extraocular extension (present/absent), and vascular invasion (present/absent).

### 2.3. Immunohistochemistry

Serial sections (4 μm) were cut from representative FFPE tumor blocks. Antigen retrieval was performed using DAKO PT Link (Agilent Technologies, Santa Clara, CA, USA) at 97 °C for 20 min in citrate buffer (pH 6.0). Automated staining was performed on a DAKO Autostainer Link 48 platform (Agilent Technologies, Santa Clara, CA, USA) using a mouse monoclonal anti-BAP1 antibody (clone C-4, 1:50, Santa Cruz Biotechnology, Santa Cruz, CA, USA) with 60 min incubation at room temperature. Endogenous peroxidase activity was blocked with 0.3% hydrogen peroxide for 10 min. The DAKO EnVision Flex Plus detection system (Agilent Technologies, Santa Clara, CA, USA) was used for visualization. Both 3-amino-9-ethylcarbazole (AEC) and 3,3′-diaminobenzidine (DAB) chromogens were employed in parallel to address the well-documented challenge of distinguishing chromogen signal from endogenous melanin pigment in pigmented tumors [21]. The AEC chromogen produces a red reaction product that is easily distinguished from the brown/black melanin pigment commonly present in UM tissue. In contrast, the brown DAB chromogen can be difficult to distinguish from melanin, especially in heavily pigmented tumors. Thus, the AEC-stained sections provide clearer visualization of BAP1-positive and -negative cells in pigmented specimens. Tissue bleaching was not performed to preserve antigenic epitopes. Hematoxylin counterstaining was applied. Appropriate positive (non-neoplastic hepatic tissue) and negative (primary antibody omission) controls were included in each run.

### 2.4. Assessment of BAP1 Expression

BAP1 nuclear expression was evaluated by light microscopy (Olympus BX-41, Olympus Corporation, Tokyo, Japan) independently by the investigator under the supervision of a senior pathologist. Expression was graded semiquantitatively as follows: (−) absent—complete loss of nuclear staining in tumor cells; (+) weak—nuclear staining in <10% of tumor cells; (++) moderate—nuclear staining in 10–50% of tumor cells; (+++) strong—nuclear staining in >50% of tumor cells.

Complete loss of nuclear BAP1 expression in tumor cells, with preserved expression in adjacent non-neoplastic stromal cells and vascular endothelium (serving as an internal positive control), was classified as BAP1 loss. For the primary statistical analysis, expression was dichotomized: BAP1 loss (categories −, +, and ++, i.e., ≤50% positive tumor cells) versus BAP1 retained (category +++ only, i.e., >50% positive tumor cells). This cutoff was selected based on the established principle that strong, diffuse nuclear staining (>50%) most reliably indicates functional BAP1 protein expression. A sensitivity analysis using an alternative cutoff (BAP1 loss defined as categories − and + only, i.e., <10% positive tumor cells, versus BAP1 retained defined as categories ++ and +++, i.e., ≥10%) was also performed to assess the robustness of findings across different thresholds.

### 2.5. Statistical Analysis

Statistical analysis was performed using IBM SPSS Statistics version 21.0 (IBM Corporation, Armonk, NY, USA). Continuous variables were described as mean ± standard deviation (SD) or median with range/interquartile range (IQR), depending on the normality of the distribution (Shapiro–Wilk test). Categorical variables were expressed as frequencies and percentages. Associations between BAP1 status and categorical clinicopathological variables were assessed using Pearson’s chi-square test or Fisher’s exact test when expected cell frequencies were <5. Continuous variables were compared between BAP1 groups using the Mann–Whitney U test.

Survival analysis was performed using the Kaplan–Meier method with log-rank (Mantel–Cox) test comparisons. Univariate and multivariate prognostic analyses were conducted using Cox proportional hazards regression, with results expressed as hazard ratios (HR) and 95% confidence intervals (CI). The proportional hazards assumption was assessed using Schoenfeld residual tests and log-log plots. Variables with *p* < 0.20 in univariate analysis were included in the multivariate model.

A priori power analysis using G*Power 3.1 (Heinrich-Heine University, Düsseldorf, Germany) confirmed >80% power for detecting large effect sizes (Cohen’s w = 0.5) with the available sample of 58 patients for overall cohort comparisons. However, due to severely imbalanced BAP1 distribution (53 versus 5 patients), the study was underpowered for meaningful comparative survival analyses between BAP1 groups, and these analyses should be considered exploratory rather than inferential. The limited number of events relative to the number of covariates constrained the use of multivariate models. No correction for multiple testing was applied due to the exploratory nature of the subgroup analyses. All tests were two-sided, with *p* < 0.05 considered statistically significant.

## 3. Results

### 3.1. Patient Demographics and Tumor Characteristics

The study cohort comprised 58 patients (30 men [51.7%] and 28 women [48.3%]) with a median age at diagnosis of 62 years (range 31–87; mean 59.8 ± 14.2 years). The most common histological type was mixed cell melanoma (36/58, 62.1%), followed by spindle cell (13/58, 22.4%) and epithelioid (9/58, 15.5%) melanoma. The mean LTD was 15.8 ± 3.72 mm (median 15.5 mm, range 10–24 mm). Ciliary body involvement was present in 8 (13.8%) tumors. Scleral involvement was observed in 49 (84.5%) cases, extraocular extension in 7 (12.1%), and vascular invasion in 25 (43.1%). The median mitotic count was 3 per mm^2^ (range 0–25). Lymphocytic infiltration was present in 11 (19.0%) specimens.

The median follow-up was 11.2 years (range 0.3–21.3; mean 10.8 ± 4.1 years). During the follow-up period, 32 (55.2%) patients died: 26 (81.3% of deaths) from metastatic UM and 6 (18.7%) from other causes. Metastatic disease occurred in 26 (44.8%) patients, with a median time to metastasis of 3.8 years (range 0.5–12.4; mean 4.2 ± 3.1 years). Comprehensive demographic and clinicopathological data are presented in Table 1.

### 3.2. BAP1 Immunohistochemical Expression

**Figure 1:**
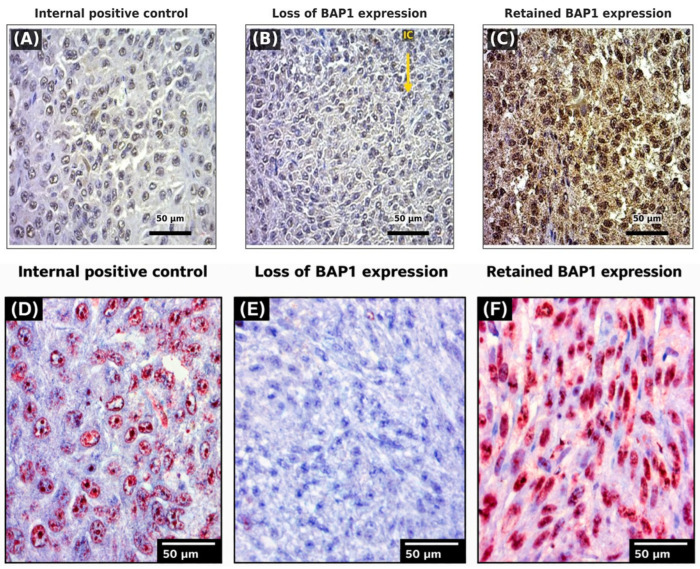
Representative immunohistochemical staining for BAP1 in uveal melanoma using DAB (panels (**A**–**C**), upper row) and AEC (panels (**D**–**F**), lower row) chromogens, with hematoxylin counterstain (scale bars = 50 µm). The brown DAB chromogen can be difficult to distinguish from endogenous melanin pigment in pigmented tumors; therefore, parallel AEC staining was performed, as the red AEC reaction product is easily distinguishable from brown/black melanin. (**A**) Internal positive control (DAB): non-neoplastic hepatic tissue showing preserved nuclear BAP1 immunoreactivity (brown). (**B**) Loss of BAP1 expression (DAB): complete absence of nuclear staining in tumor cells. The yellow arrow (IC) indicates an interspersed non-neoplastic stromal cell with preserved BAP1 nuclear staining, serving as an internal positive control. (**C**) Retained BAP1 expression (DAB): strong, diffuse nuclear immunoreactivity (brown) in tumor cells, classified as category +++ (>50% positive tumor cells). (**D**) Internal positive control (AEC): non-neoplastic hepatic tissue with preserved nuclear BAP1 immunoreactivity (red), confirming adequate antibody reactivity with the AEC detection system. (**E**) Loss of BAP1 expression (AEC): complete absence of red nuclear staining in tumor cells, showing only hematoxylin-stained nuclei visible (blue/purple), confirming true loss of BAP1. (**F**) Retained BAP1 expression (AEC): strong, diffuse red nuclear staining in tumor cells, clearly distinguishable from endogenous melanin pigment (brown/black), illustrating the advantage of AEC over DAB in pigmented uveal melanoma specimens.

**Figure 2:**
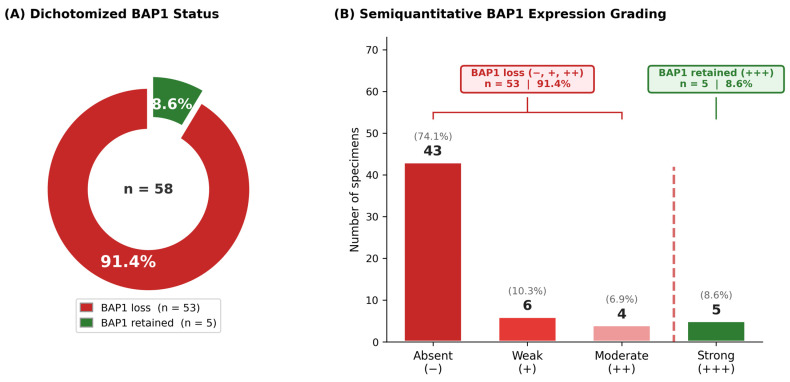
Distribution of BAP1 nuclear expression in uveal melanoma specimens (*n* = 58). (**A**) Dichotomized BAP1 status: BAP1 loss (red; *n* = 53, 91.4%) versus BAP1 retained (green; *n* = 5, 8.6%). (**B**) Semiquantitative BAP1 expression grading: absent (−) in 43 (74.1%), weak (+) in 6 (10.3%), moderate (++) in 4 (6.9%), and strong (+++) in 5 (8.6%). The red dashed line demarcates the BAP1 loss category (−, +, and ++, comprising 91.4%) from the BAP1 retained category (category +++ only, 8.6%).

Representative examples of retained and lost BAP1 expression are shown in Figure 1, with parallel DAB (panels A–C) and AEC (panels D–F) chromogen staining demonstrating the advantage of AEC in distinguishing immunoreactivity from endogenous melanin pigment. Loss of nuclear BAP1 expression was identified in 53/58 (91.4%) tumors. Only 5 (8.6%) tumors retained nuclear BAP1 expression (Figure 2A). Semiquantitative analysis revealed the following distribution: absent expression (−) in 43 specimens (74.1%), weak (+) in 6 specimens (10.3%), moderate (++) in 4 specimens (6.9%), and strong (+++) in 5 specimens (8.6%) (Figure 2B). In all positive cases, strong nuclear staining in tumor cells was accompanied by internal positive controls (stromal fibroblasts and endothelial cells).

### 3.3. Associations Between BAP1 Status and Clinicopathological Parameters

**Figure 3:**
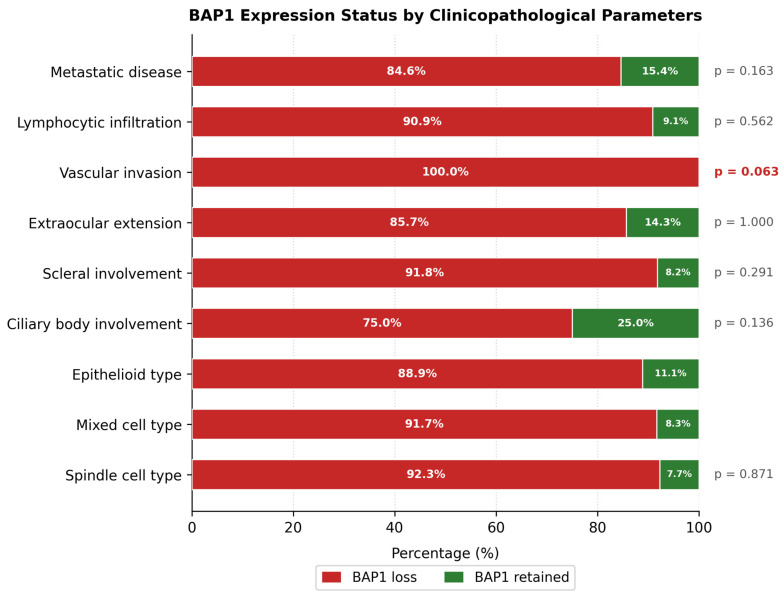
BAP1 expression status by clinicopathological parameters. Horizontal stacked bars show the proportion of patients with BAP1 loss (red) versus BAP1 retained (green). A trend toward an association was observed between BAP1 loss and vascular invasion (*p* = 0.063). No significant associations were found with histological type (*p* = 0.871), ciliary body involvement (*p* = 0.136), or metastatic status (*p* = 0.163).

A trend toward an association was observed between BAP1 loss and vascular invasion (Fisher’s exact test, *p* = 0.063): all 25 tumors (100%) with vascular invasion showed BAP1 loss, compared with 84.8% of tumors without vascular invasion. No significant associations were found between BAP1 status and sex (*p* = 0.678), age at diagnosis (*p* = 0.739), histological type (*p* = 0.871), tumor size (LTD, *p* = 0.582), ciliary body involvement (*p* = 0.136), lymphocytic infiltration (*p* = 0.562), scleral involvement (*p* = 0.291), extraocular extension (*p* = 1.000), or mitotic count (*p* = 0.412). BAP1 loss was present in 84.6% of patients who developed metastases, compared with 96.9% of those who remained metastasis-free (*p* = 0.163). Detailed results are presented in Table 2 and Figure 3.

### 3.4. Survival Analysis

#### 3.4.1. Overall Cohort Survival

**Figure 4:**
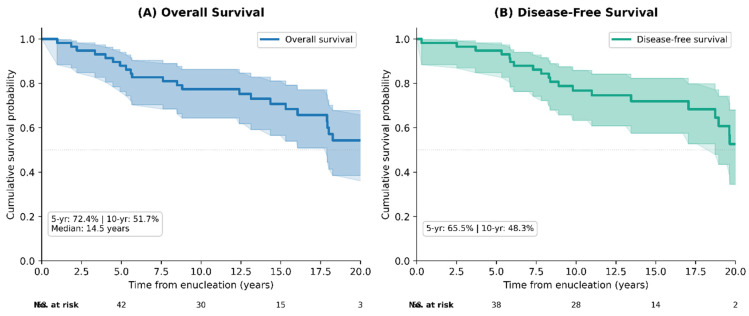
Kaplan–Meier survival curves for the entire cohort (*n* = 58). (**A**) Overall survival (OS): 5-year OS 72.4%, 10-year OS 51.7%, median OS 14.5 years. (**B**) Disease-free survival (DFS): 5-year DFS 65.5%, 10-year DFS 48.3%. Shaded areas represent 95% confidence intervals. Numbers at risk are shown below each panel.

Kaplan–Meier analysis of the entire cohort demonstrated 5-year and 10-year OS rates of 72.4% (95% CI: 58.9–82.3%) and 51.7% (95% CI: 37.8–64.0%), respectively, with a median OS of 14.5 years (95% CI: 10.3–18.7). DFS rates at 5 and 10 were 65.5% (95% CI: 51.6–76.4%) and 48.3% (95% CI: 34.5–60.8%), respectively (Figure 4).

#### 3.4.2. Survival by BAP1 Status

**Figure 5:**
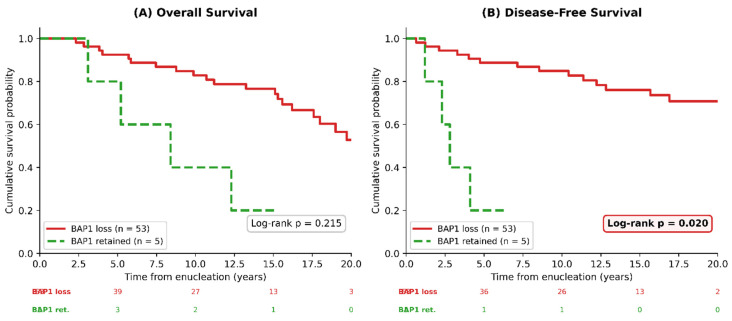
Kaplan–Meier survival curves stratified by BAP1 status. (**A**) Overall survival (OS, log-rank *p* = 0.215): no significant difference was observed between BAP1 loss (red, *n* = 53) and BAP1 retained (green dashed, *n* = 5). (**B**) Disease-free survival (DFS, log-rank *p* = 0.020): the BAP1-retained group showed a shorter DFS; this counterintuitive finding reflects a statistical artifact attributable to concurrent adverse features and the extremely small subgroup size (*n* = 5). Numbers at risk are shown below each panel.

BAP1 loss was not significantly associated with OS (log-rank test, *p* = 0.215). The median OS was 14.5 years in the BAP1-loss group and 12.3 years in the BAP1-retained group. In an exploratory analysis, BAP1 loss was nominally associated with longer DFS (log-rank *p* = 0.020): the BAP1-retained group (*n* = 5) had a median DFS of 2.3 years, while the BAP1-loss group (*n* = 53) did not reach median DFS during follow-up (Figure 5). However, this counterintuitive finding reflects a statistical artifact driven by the extremely small and unrepresentative BAP1-retained group (*n* = 5), all of whom had concurrent adverse features, and should not be interpreted biologically (see Section 4).

#### 3.4.3. Exploratory Univariate and Multivariate Analysis

The following survival analyses should be interpreted with caution due to severely imbalanced BAP1 groups (53 versus 5 patients) and the limited number of events, which render the study underpowered for prognostic inference. In univariate Cox regression for OS, epithelioid histological type showed a non-significant trend toward worse survival (HR = 2.87; 95% CI: 0.86–9.55; *p* = 0.086), whereas BAP1 loss was not significant for OS (HR = 0.58; 95% CI: 0.22–1.51; *p* = 0.264).

**Figure 6:**
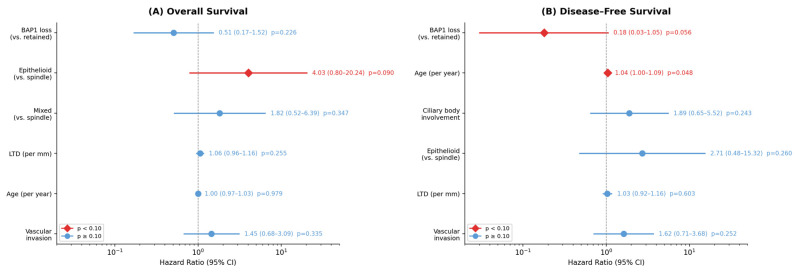
Forest plots of multivariate Cox proportional hazards regression. (**A**) Overall survival: epithelioid histology showed a trend toward significance (HR = 4.03; 95% CI: 0.80–20.24; *p* = 0.090). (**B**) Disease-free survival: patient age was the only independent predictor (HR = 1.04; 95% CI: 1.00–1.09; *p* = 0.048). BAP1 loss showed a non-significant protective association (HR = 0.18; 95% CI: 0.03–1.05; *p* = 0.056). Red diamonds indicate *p* < 0.10; blue circles indicate *p* ≥ 0.10.

In multivariate analysis for OS, including BAP1 status, histological type, LTD, age, and vascular invasion, no variable reached independent significance (Table 3, Figure 6A). Of note, the inclusion of five covariates for 32 events exceeds the recommended ratio of approximately 10 events per variable, and these results should be interpreted with caution. Epithelioid histology tended to be associated with worse OS (HR = 4.03; 95% CI: 0.80–20.24; *p* = 0.090).

For DFS, the multivariate model including BAP1 status, age, ciliary body involvement, histological type, LTD, and vascular invasion identified patient age as the only statistically significant independent predictor (HR = 1.04; 95% CI: 1.00–1.09; *p* = 0.048), indicating a 4% increase in metastatic risk per year of age at diagnosis. BAP1 loss showed a nominally protective association (HR = 0.18; 95% CI: 0.03–1.05; *p* = 0.056); however, this reflects a statistical artifact due to the extremely small BAP1-retained group (*n* = 5) and unstable hazard ratio estimates and should not be interpreted as evidence of a true biological effect (Table 3, Figure 6B). The proportional hazards assumption was satisfied for all variables (Schoenfeld test, all *p* > 0.05). Given the inclusion of six covariates in 26 DFS events, the model exceeds the recommended events-per-variable ratio, and the results should be regarded as exploratory.

### 3.5. Comparison with Published Literature

**Figure 7:**
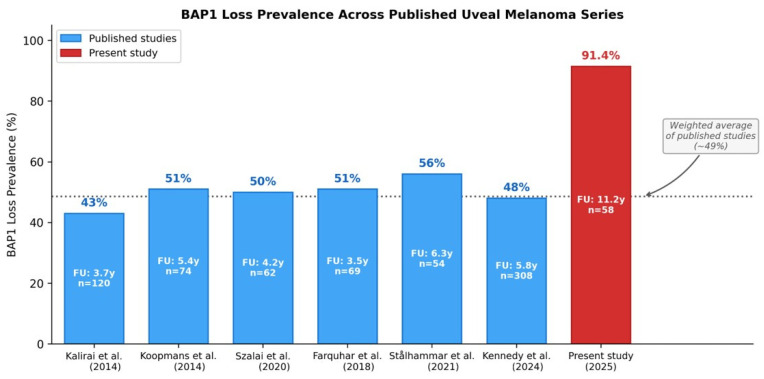
BAP1 loss prevalence across published uveal melanoma series. Bar chart comparing BAP1 loss rates from six major published IHC studies (blue) with the present study (red). Published rates ranged from 43% to 56% (weighted average ~50%, dotted line), while the present study reports 91.4%. The difference reflects the enucleation-enriched, high-risk composition of our cohort and methodological approach. Median follow-up duration for each study is indicated by the bars [11,12,13,14,15,17].

To contextualize the exceptionally high prevalence of BAP1 loss (91.4%) observed in our cohort, we compiled data from six major published BAP1 IHC studies in UM (Table 4, Figure 7). Reported BAP1 loss rates ranged from 43% to 56%, with a weighted average of approximately 50%. Thus, our observed prevalence exceeds these values by 35–48 percentage points.

## 4. Discussion

This descriptive study provides the first comprehensive IHC analysis of BAP1 expression in UM from a Croatian institution, with a median follow-up of 11.2 years–substantially longer than that of most published series. Three principal findings emerge: (1) the exceptionally high prevalence of BAP1 loss, attributable to the enucleation-based study design; (2) the severely imbalanced BAP1 distribution, which precludes meaningful prognostic analysis; and (3) the confirmation of a prolonged natural history, highlighting the need for extended patient surveillance.

### 4.1. BAP1 Loss Prevalence in the Context of Cohort Selection

The 91.4% prevalence of BAP1 loss in our cohort is the highest reported in any primary UM series and warrants careful interpretation. Published studies report BAP1 loss rates of 43–56% in primary UM specimens [11,12,13,14,15,16,17], while rates of 81–84% have been documented in metastatic lesions [22]. Our finding falls between these ranges, approaching the prevalence observed in metastatic tissue.

Several factors may contribute to this exceptionally high prevalence. While the enucleation-based study design is enriched for advanced tumors, this alone cannot fully account for the discrepancy: the Dublin series by Kennedy et al. [17] also evaluated exclusively enucleation specimens and reported only 48% BAP1 loss, while the Liverpool cohort [11] had a comparable mean LTD (16.5 mm versus 15.8 mm in our series). Additional methodological factors likely play a role. First, differences in BAP1 staining protocols, antibody clones, and scoring thresholds across institutions may significantly influence reported prevalence. In our study, we employed a threshold of >50% positive tumor cells to define BAP1 retention (category +++ only), which is more stringent than the ≥10% cutoff used in some published series. This stricter cutoff reclassifies moderate expressors (10–50%) as BAP1 loss, which accounts for a substantial portion of the observed discrepancy. Second, true geographic or population-specific differences in BAP1 loss frequency among Croatian UM patients cannot be excluded, although this hypothesis requires validation in larger cohorts. Third, our cohort characteristics—85% scleral involvement and 43% vascular invasion—indicate a particularly advanced subset even within the enucleation population.

Stålhammar et al. [23] reported that 69% of tumors in their enucleation series were AJCC stage IIb or higher, and BAP1-lost tumors had significantly greater mean volumes (2109 vs. 1552 mm^3^, *p* = 0.025). Ewens et al. [24] directly compared enucleation specimens with fine-needle aspiration biopsies and found that metastases developed in 70% of enucleated eyes versus 36% of biopsied tumors (*p* = 0.006), while the frequency of BAP1 mutations was comparable between the two groups (*p* = 0.79). These findings suggest that survival differences are primarily driven by tumor size and stage rather than BAP1 status per se, highlighting that enucleation cohorts represent a biologically distinct patient population.

A critical implication for clinical practice is that BAP1 IHC results must be interpreted in the context of the tissue acquisition method. The prognostic utility of BAP1 loss—demonstrated convincingly in large, balanced cohorts by Kennedy et al. [17] and Kalirai et al. [11]—is diminished when the reference group (BAP1-retained) is extremely small, as in our cohort (*n* = 5). This underscores the need for multi-center collaborative studies that include both enucleation and biopsy specimens to establish generalizable prognostic models.

### 4.2. The Counterintuitive Survival Finding: A Statistical Artifact

The nominally significant association between BAP1 loss and longer DFS (log-rank *p* = 0.020) directly contradicts the extensive literature establishing BAP1 loss as an adverse prognostic marker [11,12,13,14,15,16,17]. Several studies with adequately powered reference groups have consistently reported the opposite: Stålhammar et al. reported a median metastasis-free survival of 2.4 years for BAP1-lost versus 16.0 years for BAP1-retained tumors (*p* < 0.0001) [16], Koopmans et al. observed DFS of 32 versus 133 months (*p* < 0.001) [12], and Liu-Smith and Lu calculated a hazard ratio of 9.57 (95% CI: 2.82–32.5) for overall mortality with low BAP1 expression [22].

This finding represents a statistical artifact driven by three compounding factors. First, the extremely small BAP1-retained group (*n* = 5) produces unstable survival estimates with wide confidence intervals. Second, all five patients with retained BAP1 harbored independent adverse features: large tumors (4/5 with LTD > 15 mm), ciliary body involvement (2/5), and epithelioid morphology (1/5). Third, the likelihood of confounding is high when the reference group represents <10% of the total cohort. This interpretation aligns with that of Shields et al. [25], who noted that Kaplan–Meier curves in small subgroups can appear similar yet fail to reach statistical significance.

### 4.3. Extended Follow-Up and Natural History

The median follow-up of 11.2 years in our cohort provides valuable insights into the natural history of UM. Notably, metastatic events continued to occur beyond 10 years post-enucleation, with the latest metastasis documented at 12.4 years. This extended latency is consistent with the concept of tumor dormancy in UM and carries important clinical implications: current AJCC staging models and surveillance guidelines, which primarily focus on the first five years, may inadequately capture late-onset metastases. Although definitive surveillance recommendations are beyond the scope of this study, our findings suggest that follow-up limited to five years may miss a subset of metastatic events.

The 5-year and 10-year OS rates of 72.4% and 51.7% in our cohort are broadly consistent with those reported in historical enucleation series. For comparison, the COMS study reported 5-year and 10-year melanoma-specific mortality rates of approximately 16% and 34%, respectively, for medium-sized tumors [26], whereas the European EUMC multicenter study reported 5-year OS of 62% for large tumors [27]. Our intermediate outcomes likely reflect the mixed tumor-size distribution in our cohort.

### 4.4. Clinical Implications and Future Directions

The near-universal BAP1 loss in our cohort (91.4%) has important clinical implications. First, it reinforces the concept that patients requiring enucleation represent a uniformly high-risk population, for whom enrollment in adjuvant therapy trials should be prioritized. Second, it highlights that, specifically for enucleation specimens, BAP1 IHC alone offers limited discriminative value. As demonstrated in a recent comprehensive analysis of 1140 primary UM tumors across all sizes [18], molecular characterization beyond BAP1 IHC is essential for accurate prognostic stratification. Complementary prognostic markers, including chromosome 8q status, gene expression profiling, and PRAME expression [28], may further refine risk assessment in this already high-risk group [29].

The therapeutic landscape for metastatic UM is evolving rapidly. Beyond tebentafusp [6], the combination of darovasertib and crizotinib has shown promising results in the OptimUM-01 trial [30], and HDAC inhibitors targeting the BAP1–HDAC4 epigenetic axis [31] represent a biologically rational approach validated in preclinical models [32,33]. Characterization of BAP1 status in treatment-naive primary tumors, as performed in this study, provides the essential baseline data to inform future biomarker-guided therapeutic strategies.

### 4.5. Strengths and Limitations

Strengths of this study include the extended follow-up (median 11.2 years), comprehensive clinicopathological characterization, mortality data from the Croatian National Cancer Registry, and the novelty of providing the first BAP1 IHC data from a Croatian UM cohort. The use of dual chromogen systems (AEC and DAB) effectively addresses the technical challenge of melanin interference.

Limitations include the retrospective design, relatively small sample size (*n* = 58), and the highly unbalanced BAP1 distribution (91.4% loss), which precludes reliable prognostic assessment. A major limitation is the exclusive reliance on IHC without molecular validation. The absence of BAP1 mutation analysis, assessment of chromosome 3 status (e.g., monosomy 3 by FISH or other methods), and gene expression profiling classification limits both biological interpretation and comparison with genomic data. Potential discordance between IHC and molecular findings is a recognized concern: IHC detects protein loss but may not capture all functionally relevant mutations, and conversely, some mutations may not result in detectable protein loss. These molecular data were not available for the study period (2006–2016) at our institution. Additionally, the single-center design and exclusive reliance on enucleation specimens limit generalizability. Future multi-center studies incorporating both enucleation and biopsy specimens, with concurrent molecular profiling, are needed to fully characterize the prognostic utility of BAP1 across the spectrum of UM disease.

## 5. Conclusions

This descriptive study provides the first comprehensive BAP1 IHC analysis in a Croatian UM cohort, revealing an exceptionally high prevalence of BAP1 loss (91.4%) that reflects both the systematic enrichment for advanced tumors inherent in enucleation-based study designs and the stringent IHC scoring threshold employed. The severely imbalanced BAP1 distribution (53 versus 5 patients) rendered the study underpowered for prognostic inference, and the counterintuitive survival association observed is a statistical artifact that should not be interpreted biologically. The extended median follow-up of 11.2 years confirms the prolonged natural history of UM, with metastatic events occurring beyond 10 years from primary treatment. These findings underscore the critical importance of adequately sized reference groups in biomarker studies and the need for molecular validation beyond IHC alone. Future multi-center studies integrating BAP1 IHC with chromosome 3 status, gene expression profiling, and diverse treatment modalities are warranted to establish the prognostic utility of BAP1 across the full spectrum of UM disease.

## Figures and Tables

**Table 1:** Patient Demographics and Clinicopathological Characteristics (*n* = 58).

Parameter	Value
**Sex**	
Male	30 (51.7%)
Female	28 (48.3%)
**Age at diagnosis (years)**	
Mean ± SD	59.8 ± 14.2
Median (range)	62 (31–87)
**Histological type**	
Spindle cell	13 (22.4%)
Mixed cell	36 (62.1%)
Epithelioid	9 (15.5%)
**Largest tumor diameter (mm)**	
Mean ± SD	15.8 ± 3.72
Median (range)	15.5 (10–24)
**Ciliary body involvement**	8 (13.8%)
**Scleral involvement**	49 (84.5%)
**Extraocular extension**	7 (12.1%)
**Vascular invasion**	25 (43.1%)
**Mitotic count (/mm^2^)**	
Median (range)	3 (0–25)
**Lymphocytic infiltration**	11 (19.0%)
**Follow-up (years)**	
Median (range)	11.2 (0.3–21.3)
Mean ± SD	10.8 ± 4.1
**Outcome**	
Alive	26 (44.8%)
Dead—uveal melanoma	26 (44.8%)
Dead—other causes	6 (10.3%)
Metastatic disease	26 (44.8%)
**Time to metastasis (years)**	
Median (range)	3.8 (0.5–12.4)
Mean ± SD	4.2 ± 3.1

SD, standard deviation. Bold text and blue color are used to emphasize key values within the table.

**Table 2:** Associations of BAP1 Status with Clinicopathological Parameters.

Parameter	BAP1 Loss (*n* = 53)	BAP1 Retained (*n* = 5)	*p* -Value
**Sex**			0.678 ^a^
Male	28 (52.8%)	2 (40.0%)	
Female	25 (47.2%)	3 (60.0%)	
**Age at diagnosis** (years), median (range)	62 (31–87)	58 (42–78)	0.739 ^b^
**Histological type**			0.871 ^a^
Spindle cell	12 (22.6%)	1 (20.0%)	
Mixed cell	33 (62.3%)	3 (60.0%)	
Epithelioid	8 (15.1%)	1 (20.0%)	
**LTD** (mm), mean ± SD	15.9 ± 3.8	15.0 ± 3.2	0.582 ^b^
**Ciliary body involvement**	6 (11.3%)	2 (40.0%)	0.136 ^a^
**Scleral involvement**	45 (84.9%)	4 (80.0%)	0.291 ^a^
**Extraocular extension**	6 (11.3%)	1 (20.0%)	1.000 ^a^
**Vascular invasion**	25 (47.2%)	0 (0.0%)	**0.063 ^a^**
**Mitotic count** (/mm^2^), median (range)	3 (0–25)	2 (0–8)	0.412 ^b^
**Lymphocytic infiltration**	10 (18.9%)	1 (20.0%)	0.562 ^a^
**Metastatic disease**	22 (41.5%)	4 (80.0%)	0.163 ^a^

LTD, largest tumor diameter; SD, standard deviation. ^a^ Fisher’s exact test; ^b^ Mann–Whitney U test. Red bold indicates *p* < 0.10. Bold text and blue color are used to emphasize key values within the table.

**Table 3:** Exploratory Univariate and Multivariate Cox Proportional Hazards Regression Analysis.

Variable	Univariate HR (95% CI)	*p* -Value	Multivariate HR (95% CI)	*p* -Value
**Overall Survival**				
BAP1 loss (vs. retained)	0.58 (0.22–1.51)	0.264	0.64 (0.20–2.04)	0.448
Epithelioid type (vs. spindle/mixed)	2.87 (0.86–9.55)	0.086	4.03 (0.80–20.24)	0.090
LTD (per mm)	1.05 (0.95–1.16)	0.332	1.03 (0.91–1.17)	0.616
Age (per year)	1.02 (0.99–1.05)	0.185	1.02 (0.98–1.06)	0.294
Vascular invasion	1.48 (0.72–3.04)	0.286	1.25 (0.55–2.85)	0.596
**Disease-Free Survival**				
BAP1 loss (vs. retained)	0.27 (0.09–0.82)	**0.020**	0.18 (0.03–1.05)	0.056
Age (per year)	1.03 (1.00–1.06)	0.088	**1.04 (1.00–1.09)**	**0.048**
Ciliary body involvement	1.96 (0.74–5.17)	0.175	1.73 (0.56–5.39)	0.342
Epithelioid type (vs. spindle/mixed)	1.68 (0.58–4.87)	0.340	1.52 (0.42–5.54)	0.523
LTD (per mm)	1.04 (0.93–1.15)	0.506	1.01 (0.88–1.15)	0.927
Vascular invasion	1.62 (0.75–3.49)	0.221	1.34 (0.55–3.28)	0.524

HR, hazard ratio; CI, confidence interval; LTD, largest tumor diameter. Red bold indicates *p* < 0.05. Variables with *p* < 0.20 in univariate analysis were included in the multivariate model.

**Table 4:** Comparison of BAP1 Loss Prevalence Across Published Uveal Melanoma IHC Studies.

Study	Year	*n*	Cohort Type	BAP1 Loss *n* (%)	Median FU (Years)
Kalirai et al. [11]	2014	120	Enucleation	52 (43%)	3.7
Koopmans et al. [12]	2014	74	Mixed (enucl. + biopsy)	38 (51%)	5.4
Szalai et al. [13]	2020	62	Enucleation	31 (50%)	4.2
Farquhar et al. [14]	2018	69	Enucleation	35 (51%)	3.5
Stålhammar et al. [15]	2021	54	Enucleation	30 (56%)	6.3
Kennedy et al. [17]	2024	308	Enucleation	148 (48%)	5.8
**Present study**	**2025**	**58**	**Enucleation**	**53 (91.4%)**	**11.2**

IHC, immunohistochemistry; FU, follow-up; enucl., enucleation. The present study is highlighted.

## Data Availability

The data presented in this study are available upon reasonable request from the corresponding author. Data are not publicly available due to patient privacy considerations.
